# CFTR Deletion Confers Mitochondrial Dysfunction and Disrupts Lipid Homeostasis in Intestinal Epithelial Cells

**DOI:** 10.3390/nu10070836

**Published:** 2018-06-27

**Authors:** Marie L. Kleme, Alain Sané, Carole Garofalo, Ernest Seidman, Emmanuelle Brochiero, Yves Berthiaume, Emile Levy

**Affiliations:** 1 Research Centre, CHU Sainte-Justine, 3175 Sainte-Catherine Rd., Montreal, QC H3T 1C5, Canada; dockleme@yahoo.fr (M.L.K.); sanealaintheo@gmail.com (A.S.); carole.garofalo@recherche-ste-justine.qc.ca (C.G.); Emmanuelle.Brochiero@umontreal.ca (E.B.); 2 Department of Nutrition, Faculty of Medicine, Université de Montréal, 2405 Sainte-Catherine Rd., Montreal, QC H3T 1A8, Canada; 3 Division of Gastroenterology, Faculty of Medicine, IBD Laboratory, Research Institute, McGill University Health Center, McGill University, Montreal, QC H3G 1A4, Canada; Ernest.Seidman@McGill.ca; 4 Montreal Clinical Research Institute, Montreal, QC H2W 1R7, Canada; yves.berthiaume@ircm.qc.ca

**Keywords:** cystic fibrosis, fatty acid oxidation, OXPHOS, oxidative stress, apoptosis, lipid metabolism, apolipoprotein biogenesis, lipoprotein secretion

## Abstract

Background: Cystic Fibrosis (CF) is a genetic disease in which the intestine exhibits oxidative and inflammatory markers. As mitochondria are the central source and the main target of reactive oxygen species, we hypothesized that cystic fibrosis transmembrane conductance regulator (CFTR) defect leads to the disruption of cellular lipid homeostasis, which contributes to mitochondrial dysfunction. Methods. Mitochondrial functions and lipid metabolism were investigated in Caco-2/15 cells with CFTR knockout (CFTR^-/-^) engineered by the zinc finger nuclease technique. Experiments were performed under basal conditions and after the addition of the pro-oxidant iron-ascorbate (Fe/Asc) complex. Results. Mitochondria of intestinal cells with CFTR^-/-^, spontaneously showed an altered redox homeostasis characterised by a significant decrease in the expression of PPARα and nuclear factor like 2. Consistent with these observations, 8-oxoguanine-DNA glycosylase, responsible for repair of ROS-induced DNA lesion, was weakly expressed in CFTR^-/-^ cells. Moreover, disturbed fatty acid β-oxidation process was evidenced by the reduced expression of CPT1 and acyl-CoA dehydrogenase long-chain in CFTR^-/-^ cells. The decline of mitochondrial cytochrome c and B-cell lymphoma 2 expression pointing to magnified apoptosis. Mitochondrial respiration was also affected as demonstrated by the low expression of respiratory oxidative phosphorylation (OXPHOS) complexes and a high adenosine diphosphate/adenosine triphosphate ratio. In contrast, the FAS and ACC enzymes were markedly increased, thereby indicating lipogenesis stimulation. This was associated with an augmented secretion of lipids, lipoproteins and apolipoproteins in CFTR^-/-^ cells. The addition of Fe/Asc worsened while butylated hydroxy toluene partially improved these processes. Conclusions: CFTR silencing results in lipid homeostasis disruption and mitochondrial dysfunction in intestinal epithelial cells. Further investigation is needed to elucidate the mechanisms underlying the marked abnormalities in response to CFTR deletion.

## 1. Introduction

Cystic Fibrosis (CF) is a lethal genetic disease in Caucasians [1]. It is due to mutations in the CFTR gene, which encodes for the cAMP-activated Cl^−^-and HCO_3_^−^-transporting channels expressed in epithelial cells of many organs [2]. This ATP-binding cassette transporter works not only as an essential anion regulator, but it also participates in the fluidity of glandular secretions [2]. In addition to facilitating the mobilization of native anionic substrates, CFTR promotes the transport of glutathione, the first line of cellular antioxidant defense. Glutathione (GSH) is the most important actor in the control of thiol redox status [3] and is involved in free radical scavenging, metal chelation and DNA protection [4]. Many studies reported GSH deficiency in CF in association with intestinal malabsorption of fat and antioxidant lipovitamins, thereby contributing to weak protection against oxidative stress (OxS) in CF. Moreover, OxS in CF may result from endoplasmic reticulum stress, airway chronic neutrophilia and impaired antioxidant defense because of intestinal transport disorders [5,6]. In addition, mitochondria in CF exhibit several abnormalities, suggesting an oxidative impairment [7]. Finally, peroxisome proliferator activated receptor gamma coactivator-1-alpha (PGC-1α) could be impaired in CF since it represents the key regulator of mitochondria biogenesis, a control protein for the major transcription factors involved in β-oxidation, Krebs cycle and oxidative phosphorylation [8], and a central modulator of OxS through the enhancement of reactive oxygen species (ROS) detoxification enzyme [9].

OxS and inflammation co-exist in various disorders, and the two processes are recognized as major etiological factors in CF pathophysiology in the lungs and pancreas [10,11,12]. Although inflammation is triggered by airway chronic infection and essential fatty acid (FA) deficiency (leading to the elevation of pro-inflammatory FA) [13,14], dysfunctional CFTR seems to be directly involved in the activation of macrophage, modulation of cytokine production, and stimulation of nuclear factor kappa B, a powerful transcription factor endowed with the capacity of regulating many genes of innate and adaptive immunity, as well as inflammation [15].

The gastrointestinal tract of CF patients is often characterized by meconium ileus, distal intestinal obstruction, accumulation of viscous and sticky mucus, bacterial overgrowth, ileal hypertrophy and villous atrophy, augmented intestinal permeability, chronic diarrhea, fat malabsorption, reduced secretion of sodium bicarbonate, low duodenal pH, and bile acid precipitation [16]. Despite these severe gastrointestinal manifestations, only a few groups of investigators have focused on the study of the processes of inflammation and OxS in the gut of CF subjects. Our recent study showed that CFTR deletion in intestinal Caco-2/15 cells resulted in a spontaneously weakened antioxidant defense in association with lipid peroxidation and inflammation [17]. In addition, these differentiated cells were more sensitive to pro-oxidant and pro-inflammatory stimuli [6]. Unfortunately, the definitive mechanistic evidence of the link between the primary CF defect and abnormalities in intestinal redox balance and susceptibility to inflammation has yet to be demonstrated. The purpose of the present work is to test the following hypotheses: (i) as oxidant/anti-oxidant imbalance has been observed in the intestine and since mitochondria in various organs are the primary cellular sources and sensitive targets of ROS [18], we surmise that CFTR deletion affects mitochondrial redox homeostasis; (ii) consequently, many mitochondrial functions may be disturbed, including respiration, FA β-oxidation, energy production and apoptosis; and (iii) conversely, cellular lipid synthesis and transport would be enhanced.

## 2. Materials and Methods

### 2.1. CFTR-Knockout in Caco-2/15 Cell Line

The CFTR deletion in the Caco-2/15 cell line (CFTR^-/-^) was generated by the zinc finger nuclease (ZFN) procedure as previously described [17]. Briefly, intestinal Caco-2/15 cells were grown at 37 °C with 95% humidity and 5% CO_2_ in Eagle’s minimum essential medium (EMEM) supplemented with 10% decomplemented foetal bovine serum, 1% Penicillin-Streptomycin and 1% non-essential amino acids (NEAA, all reagents from GIBCO- BRL, Grand Island, NY, USA). At 95% confluence, according to the procedure provided by Sigma-Aldrich (St Louis, MO, USA), the cells were transfected with two ZFN plasmid constructs targeted to human CFTR gene (CKOZFN1722). These ZFNs bind to a specific locus on CFTR sequence and cause a double-strand break that is ultimately repaired in the cell by the process of non-homologous end joining to generate precisely targeted genomic edits resulting in cell lines with specific gene disruption. The integrity of cells was monitored by assessment of villin protein expression whereas differentiation was determined by the measurement of sucrase activity [19,20]. The physical barrier function was characterised by transepithelial electric resistance measurement and occludin protein expression [21]. The untransfected intestinal epithelial Caco-2/15 cells served as the control (CTL).

### 2.2. Cell Culture

CTL and CFTR^-/-^ cells were cultured at 37 °C with 95% humidity and 5% CO_2_, at a density of 5×10^6^/Petri dish containing 10 mL EMEM supplemented with 1% penicillin-streptomycin, 1% NEAA and 5% decomplemented foetal bovine serum. The culture medium was refreshed every three days, and the cells were used fifteen days post confluence when they were highly differentiated and suitable for experiments.

### 2.3. Isolation of Mitochondria

The mitochondrial fraction was isolated from Caco-2/15 cell monolayers by differential centrifugation according to our previously reported method [7,22,23,24]. Briefly, after homogenization in sucrose extraction buffer (0.25 M, pH 7.38), a light centrifugation (1000× *g* × 10 min at 4 °C) was performed to sediment nuclei and cell debris. The resulting supernatant was subsequently centrifuged twice (10,000× *g* × 10 min at 4 °C) to separate mitochondria. The crude mitochondrial pellet was then resuspended in sucrose buffer and used for further experiments. The protein content of the mitochondrial suspensions was determined by Bradford assay (BioRad, Mississauga, ON, Canada).

### 2.4. Induction of OxS

Fifteen days post confluence, the CTL and CFTR^-/-^ cells were washed twice with PBS and incubated in serum-free EMEM supplemented with 1% penicillin-streptomycin and 1% NEAA for 18 hours in the presence or absence of the antioxidant butylated hydroxy toluene (BHT, 0.5 mM) (Sigma-Aldrich, St Louis, MO, USA). At the end of this incubation period (18 h), the medium was removed and cells were cultured with a mixture of 200 μM iron (II) sulphate heptahydrate (Sigma-Aldrich, St Louis, MO, USA) and 2 mM ascorbate (Sigma-Aldrich, St Louis, MO, USA) for 6 h at 37 °C to induce OxS. This strong oxygen radical-generating system was employed to challenge cells and to evaluate their capacity to respond to an external pro-oxidant stimulus.

### 2.5. Protein Expression Analysis by Immunoblotting

Protein samples (30 μg) were denatured for 10 min in a buffer containing SDS and mercaptoethanol. They were separated on a 12% SDS-polyacrylamide gel according to protein molecular weights and were electroblotted onto nitrocellulose or PVDF membranes. Defatted milk proteins were used to block nonspecific sites of the membranes before adding primary antibodies: rabbit polyclonal anti-peroxisome proliferator activated receptor gamma coactivator-1-alpha (PGC-1α, 100 kDa, 1:1000, Abcam, Cambridge, MA, USA); rabbit anti- nuclear factor (erythroid-derived 2)-like 2 (Nrf-2, 68 kDa, 1:1000, Abcam, Cambridge, MA, USA); rabbit polyclonal anti-8-oxoguanine-DNA glycosylase (OGG1, 39 kDa, 1:1000, Novus biologicals, Oakville, ON, Canada); mouse anti-cytochrome c (15 kDa, 1:1000, Novus biologicals, Oakville, ON, Canada); rabbit polyclonal anti-Bcl-2 (25 kDa, 1:1000, Abcam Cambridge, MA, USA); rabbit monoclonal anti-carnitine palmitoyltransferase-1 A (CPT1A, 88 kDa, 1:1000, Cell Signaling, Beverly, MA, USA), rabbit polyclonal anti-Acyl-CoA dehydrogenase long-chain (ACADL, 45 kDa, 1:1000, ThermoFisher scientific, Burlington, ON, Canada); Total OXPHOS Human WB antibody cocktail (CV 54 kDa, CIII 48 kDa, CII 29 kDa, CIV 22 kDa, CI 18 kDa, 1:250, Abcam Cambridge, MA, USA); mouse anti-β-actin (42 kDa, 1:250,000, Sigma-Aldrich, St Louis, MO, USA); rabbit monoclonal anti-fatty acid synthase (FAS, 273 kDa, 1:1000, Cell Signaling, Beverly, MA, USA); rabbit anti-Acetyl CoA carboxylase (ACC, 280 kDa, 1:1000, Cell signaling, Beverly, MA, USA) . The relative amount of primary antibody was detected with species-specific horseradish peroxidase-conjugated secondary antibody (Jackson Laboratory, Bar Harbor, ME, USA). Expression of β-actin served as a reference protein to confirm equal loading. Molecular size markers (BLUeye/PageRuler prestained protein ladder, ThermoFisher scientific, Burlington, ON, Canada) were concomitantly loaded on gels. Blots were developed and the protein mass was quantitated using an HP Scanjet scanner equipped with a transparency adapter and the UNSCAN-IT gel 6.1 software (Silk Scientific, Orem, UT, USA). Importantly, even if identical protein amounts of tissue homogenates were applied, the β-actin protein was used to confirm equal loading on SDS-PAGE. Its expression was evidenced after stripping the blot and re-probing with its specific antibody.

### 2.6. RNA Isolation and RT-PCR

Total RNA was extracted from differentiated CTL and CFTR^-/-^ Caco-2/15 cells using QIAzol lysis reagent (Invitrogen, Thermo Fisher scientific, Burlington, ON, Canada) and reverse transcribed to generate cDNA. This was amplified by PCR using Taq polymerase (Feldan Bio, Quebec, QC, Canada) according to the manufacturer’s instructions. GAPDH (as internal control) and CFTR as described previously [17].

### 2.7. Fatty Acid β-Oxidation

Cells were rinsed twice with PBS (Gibco, Thermo Fisher scientific, Burlington, ON, Canada) before being pre-incubated with serum-free EMEM for 1 h in flasks fitted with central wells, suspended through an air-tight rubber bung. Cells were supplemented with a solution containing 0.90 µCi U-(C^14^)-palmitic acid (Perkin Elmer, 850 mCi/mmol), 250 μM unlabeled palmitic acid (Sigma-Aldrich, St Louis, MO, USA) and 2 mL serum-free EMEM and 10 µL/mL of each anti-protease (PMSF, Leupeptin and Pepstatin) for 18 h while NaOH was added to each central well to trap released (^14^C)-CO_2_, and (^14^C)-oxidized to (^14^C)-CO_2_ was measured as described previously [25]. Briefly, (^14^C)-CO_2_ was driven from medium by adding 300 μL of 0.7N hydrochloric acid and trapped in the central well saturated with 10× hyamine hydroxide. Flasks were shaken for a further 45-min period at room temperature to increase trapping of (^14^C)-CO_2_, after which the center wells were placed in plastic vials, dark-adapted overnight, and assayed for radioactivity in a liquid scintillation counter.

### 2.8. Measurement of Mitochondrial ADP/ATP Ratio

The adenosine diphosphate/adenosine triphosphate (ADP/ATP) ratio was measured by bioluminescence assay using the Enzlight^™^ ADP/ATP ratio assay kit (ELDT-100) (BioAssay Systems, Hayward, CA, USA) as described previously [7,22,23,24]. Results were normalized according to the protein content of the mitochondria.

### 2.9. Lipid and Lipoprotein Assessment

Specifically, for these experiments, cells were seeded at a density of 1×10^6^/well in six-well polycarbonate Transwell filter inserts plates (Costar Cambridge, MA, USA) containing 2.5 mL of culture medium in the basolateral compartment and 15 mL in the apical one. The culture medium was refreshed every 3 days. Fifteen days after seeding, cells were washed twice with PBS and supplied with 2.5 mL serum-free EMEM in the basolateral chamber and 1.5 mL serum-free EMEM containing (BSA/oleic acid) (1:5, mol:mol, pH 7.4) in the apical chamber. BHT (0.5 mM) was added to serve as an antioxidant when needed. After 18 h, the culture medium was discarded and replaced with fresh 2.5 mL serum-free EMEM and 1.5 mL radiolabeled (^14^C) oleic acid (0.45 μCi) (53 mCi/mmol; Amersham, Oakville, ON, Canada) in the basolateral and the apical compartments, respectively. Fe/Asc (200 μM/2 mM) with or without BHT (0.5 mM) was added apically. After a 24 h-incubation, media were collected from the two chambers and cells were washed twice with cold PBS and scraped in 1 mL of lysis buffer (1× TBS, pH 7.4; 5 mM EDTA; 0.1% SDS; 1% Triton ×100; 0.5% Sodium deoxycholate) containing 10 μL/mL of each anti-protease (PMSF, Leupeptin and Pepstatin).

### 2.10. Lipid Extraction

Lipids were extracted from the basolateral medium with chloroform/methanol (*v*/*v*, 2:1). After overnight shaking at 4 °C, saline was added and the samples were centrifuged (2500 rpm for 15 min). Then, the lower phase was evaporated. The esterified lipids from the precursor (^14^C)-oleic acid were separated by TLC plates using the solvent mixture hexane-ether-acetic acid (80:20:3, *v*/*v*/*v*). The area of triglycerides (TG), cholesteryl esters (CE) and phospholipids (PL) was scratched off the TLC plates and radioactivity contained in the silica powder was counted by scintillation counting (Beckman Coulter, Fullerton, CA, USA), using the scintillation liquid EcoLite (MP Biomedicals, ThermoFisher scientific, Burlington, ON, Canada). Results were expressed as dpm per milligram of cell protein, quantified by the Bradford method [26,27,28].

### 2.11. Lipid Carrier

Blood was drawn 3 h after the ingestion of a high-fat meal by a human volunteer. It was centrifuged to remove red blood cells, and the postprandial plasma supplemented with 1 mM of aprotinin and 0.1% of sodium azide served as a carrier for the synthesis of TG-rich lipoproteins as described previously [29].

### 2.12. Isolation of Lipoproteins

Newly synthesized lipoproteins were separated from the basolateral medium supplemented with anti-proteases and to which plasma lipid carrier was added (4:1, *v*/*v*) to efficiently isolate de novo produced lipoproteins. Isolation was performed by sequential ultracentrifugation using a TL-100 ultracentrifuge as per our usual technique [30,31,32]. Briefly, chylomicrons (CM)s were isolated after ultracentrifugation (25,000 rpm for 40 min). The lower phase was used to isolate successively very low-density lipoprotein (VLDL; 1.006 g/mL) and LDL (1.063 g/mL) at 100,000 rpm for 2.30 h with a tabletop ultracentrifuge 100.4 rotor at 4 °C. Then, the density of LDL infranatant was adjusted to 1.21 g/mL before centrifuging at 100,000 rpm for 6.5 h to efficiently isolate HDL. Each lipoprotein fraction was exhaustively dialyzed at 4 °C for 24 h into a solution contained 0.15 M NaCl and 0.001 M EDTA. The radioactivity incorporated into each lipoprotein was then assessed and reported as disintegrations per minute per milligram of cellular protein.

### 2.13. De Novo Apolipoproteins Synthesis

The synthesis and secretion of apolipoproteins (apos) were determined as described previously [33]. Briefly, Caco-2/15 cells were incubated for a period of 24 h with 67 μCi/mL of (^35^S)-methionine in serum and methionine-free DMEM (Gibco, ThermoFisher scientific, Burlington, ON, Canada) containing NEAA and puromycin (2 μg/mL). After the incubation period, cells and basolateral medium were collected and antiproteases were added at a concentration of 1 mM. Cells were homogenized in the same lysis buffer as above. Excess amounts of antibodies against apos were added to cells along with A/G protein beads (Calbiochem, La Jolla, CA, USA) and incubated overnight at 4 °C. The immunoprecipitates were washed extensively with lysis buffer and analyzed on a 4–15% acrylamide gradient with a 3% stacking gel. Bands corresponding to all apos were excised off the gel and counted after an overnight incubation with 1 mL of BTS-450 (Beckman Coulter, Fullerton, CA, USA) and a 48-h incubation in 10 mL of liquid scintillation fluid (Ready Organic, Beckman Coulter, Fullerton, CA, USA). Results are reported as the ratio of disintegrations per minute of apos per milligram of protein. 

### 2.14. Statistical Analysis

All values are expressed as the mean ± standard error of the mean (SEM) of at least 3 different experiments carried out in triplicate. Data were analyzed by the two-tailed Student’s t test or one-way ANOVA using PRISM 6.0 (GraphPad Software). Differences were considered significant at *p* < 0.05.

## 3. Results

**Figure 1:**
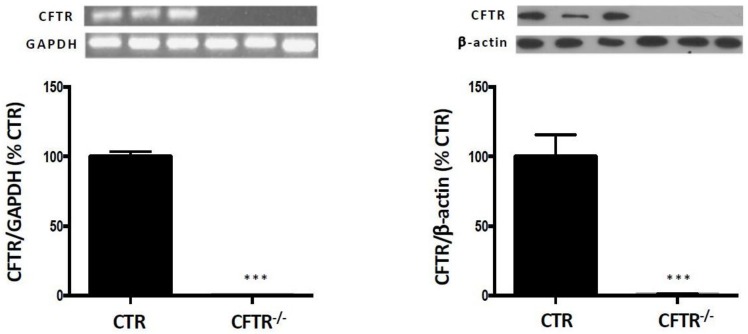
CFTR gene and protein expression in control and CFTR^-/-^ Caco-2/15 cells. Knockout of the CFTR gene by the ZFN technology resulted in full deletion of mRNA and protein expression, which were assessed by RT-PCR and Western blot, respectively. The CFTR^-/-^ cell line is thus appropriate to mimic the homozygous CF phenotype. Values are means ± standard error of the mean (SEM) of 3 independent experiments. *** *p* < 0.001 vs. CTR cells. CTR: control cells; ZFN: zinc finger nuclease.

Before initiating the series of planned experiments, we validated the effectiveness of ZFN to generate total CFTR suppression in Caco-2/15 cells. qPCR and western blot confirmed the full deletion of CFTR gene and protein expression, respectively [17] (Figure 1). Appropriate precautions have been taken to ensure that the cell integrity was preserved following genetic manipulation. Confirmation was obtained by testing trypan blue exclusion, transepithelial resistance, sucrase activity, villin protein expression and occludin protein expression (Table 1).

### 3.1. Mitochondrial Antioxidant Defense

**Figure 2:**
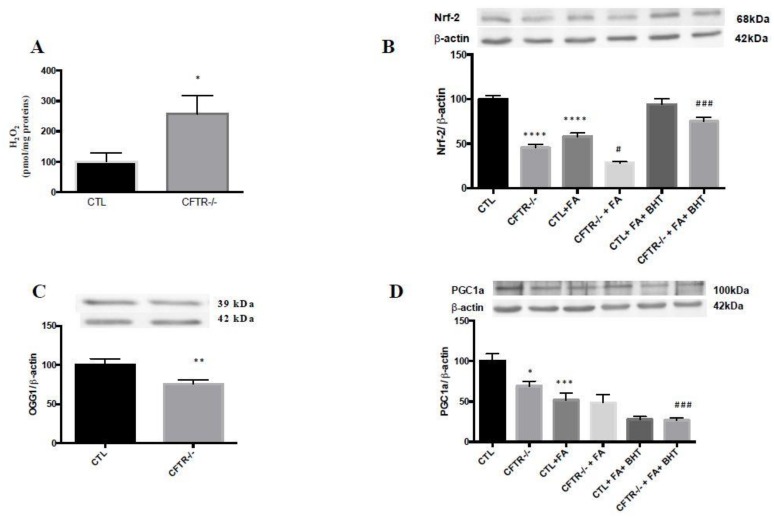
Impact of CFTR deletion on mitochondrial pro-oxidant and antioxidant defense in control and CFTR^-/-^ cells. **A**fter cellular differentiation, Caco-2/15 cells were harvested, trypsinized and homogenized. (**A**) Mitochondrial H_2_O_2_ was measured by a bioluminescence assay whereas Western blot served to estimate the protein mass of (**B**) nuclear factor (erythroid-derived 2)-like2 (Nrf2) in homogenate, (**C**) 8-oxoguanine DNA glycosylase (OGG1) in mitochondria, and (**D**) peroxisome proliferator activated receptor gamma coactivator-1-alpha (PGC-1α) in homogenate. Values are means ± SEM of 3–5 independent experiments, each done in triplicate. * *p* < 0.05, ** *p* < 0.01, *** *p* < 0.001, **** *p* < 0.0001 vs. CTL cells; # *p* < 0.05, ### *p* < 0.001 vs. CFTR^-/-^ cells. CTR: control cells; FA: iron/ascorbate (200 μM/0.5 mM); BHT: Butylated hydroxy toluene (0.5 mM).

Since our previous studies demonstrated that the absence of functional CFTR in Caco-2/15 cells promoted exaggerated pro-oxidative response [17], we evaluated the antioxidant status in mitochondria, which are considered as the central source of ROS in different organs [18]. Assessment of mitochondrial H_2_O_2_ showed increased levels in CFTR^-/-^ compared to CTL cells (Figure 2A). Conversely, there was a significant decrease in the protein expression of Nrf2, a critical transcription factor that upregulates the expression of many antioxidant genes (Figure 2B). In addition, CFTR^-/-^ Caco-2/15 cells exhibited a marked decline in the protein expression of mitochondrial OGG1, responsible for repair of ROS-induced DNA lesions in humans, [34] (Figure 2C) and in PGC-1α, the master mitochondrial biogenesis regulator and ROS suppressor [8,35] (Figure 2D). These results highlight the link between CFTR ablation and mitochondrial pro-oxidant/oxidant imbalance under basal conditions. The addition of Fe/Asc led to a further decrease of these values and the pre-incubation with BHT improved the level of Nrf2 but not PGC-1α, assessed in cell homogenates (Figure 2).

### 3.2. Fatty Acid β-Oxidation

**Figure 3:**
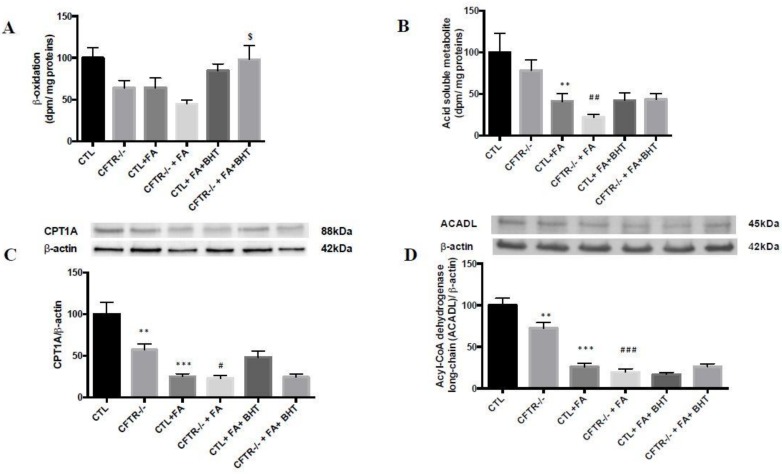
Influence of CFTR suppression on mitochondrial fatty acid β-oxidation and related regulatory enzymes. At the end of 15 days of differentiation, Caco-2/15 cells were harvested, trypsinized and resuspended. Thereafter, cells were incubated with palmitate. β-oxidation was monitored by quantification of (**A**) (^14^C)-palmitate oxidized to (^14^C)-CO_2_ and (^14^C)-acid-soluble products. (**B**) Complex II (CII). Mitochondrial protein mass of (**C**) carnitine palmitoyl transferase 1 A (CPT1A) and (**D**) acyl-coA dehydrogenase long chain (ACADL) was analyzed by western blotting. Values are means ± SEM of 3 independent experiments, each performed in triplicate. ** *p* < 0.01, *** *p* < 0.001 vs. CTL cells; # *p* < 0.05, ## *p* < 0.01, ### *p* < 0.001 vs. CFTR^-/-^ cells; $ *p* < 0.05 vs. CFTR^-/-^ + FA cells. CTR: control cells; FA: iron/ascorbate (200 μM/0.5 mM); BHT: Butylated hydroxy toluene (0.5 mM).

To test the impact of CFTR deletion on mitochondrial oxidative capacity, we incubated intestinal cells with (U-^14^C)-palmitic acid and, at the end of the incubation period, (^14^C)-CO_2_ production was estimated. As illustrated in Figure 3A, the catabolism of the labeled palmitate was significantly decreased (36%, *p* < 0.05) in CFTR^-/-^ Caco-2/15 cells. Similarly, the CFTR deletion reduced (U-^14^C)-palmitate oxidation into (^14^C)-acid-soluble products (22%, *p* < 0.05) (Figure 3B). In addition, cell treatment with Fe/Asc further reduced the FA β-oxidation while the pre-incubation with BHT attenuated Fe/Asc-induced metabolite fall and to a lesser degree in CFTR^-/-^ cells (Figure 3A).

These results prompted us to appraise the mitochondrial expression of CPT1 and ACADL since these proteins are rate-controlling enzymes of the FA β-oxidation pathway. Our results demonstrated that under basal conditions, CPT1A expression was lower in CFTR^-/-^ (43%, *p* < 0.01) than in CTL cells (Figure 3C). When Fe/Asc was added, the expression was further depressed while BHT did not significantly restore CPT1A protein expression in both CTL and CFTR^-/-^ cells. (Figure 3C). Without any treatment, the protein expression of ACADL in CFTR^-/-^ cells significantly declined (28%, *p* < 0.01) compared to CTL cells (Figure 3D). Under pro-oxidant conditions triggered by Fe/Asc, ACADL protein mass was even significantly depressed and BHT failed to re-establish it (Figure 3D).

### 3.3. Oxidative Phosphorylation

**Figure 4:**
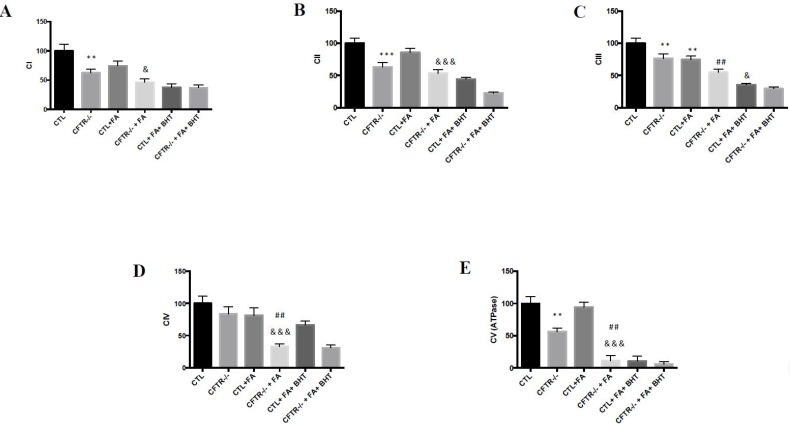
Effect of CFTR abrogation on mitochondrial oxidative phosphorylation. After 15 days differentiation, Caco-2/15 cells were trypsinized and mitochondria were isolated by ultracentrifugation to assess the protein mass by western blotting of (**A**) respiratory chain complex I (CI); (**B**) complex II (CII); (**C**) complex III (CIII); (**D**) complex IV (CIV); and (**E**) complex V (CV). Values are means ± SEM of 5 independent experiments, each performed in triplicate. ** *p* < 0.01, *** *p* < 0.001 vs. CTL cells; ## *p* < 0.01 vs. CFTR^-/-^ cells; & *p* < 0.05, &&& *p* < 0.001 vs. CTL + FA cells. CTR: control cells; FA: iron/ascorbate (200 μM/0.5 mM); BHT: Butylated hydroxy toluene (0.5 mM).

To determine whether the suppression of CFTR affects oxidative phosphorylation (OXPHOS), a central metabolic pathway in mitochondria, the respiratory chain complexes were evaluated. Their mitochondrial protein expression, examined by immunoblotting, revealed a significant decrease in complex I (CI), complex II (CII), complex III (CIII) and complex (V) in CFTR^-/-^ cells under basal conditions (Figure 4A–C,E). Complex IV (CIV) protein expression was only slightly reduced (Figure 4D). When Fe/Asc was added, the protein expression of all the complexes was further decreased particularly in CFTR^-/-^ cells compared to CTL cells (Figure 4). Pre-incubation of cells with BHT failed to normalize mitochondria respiratory chain complexes expression in both CFTR^-/-^ and CTL cells (Figure 4).

### 3.4. ADP/ATP Ratio

**Figure 5:**
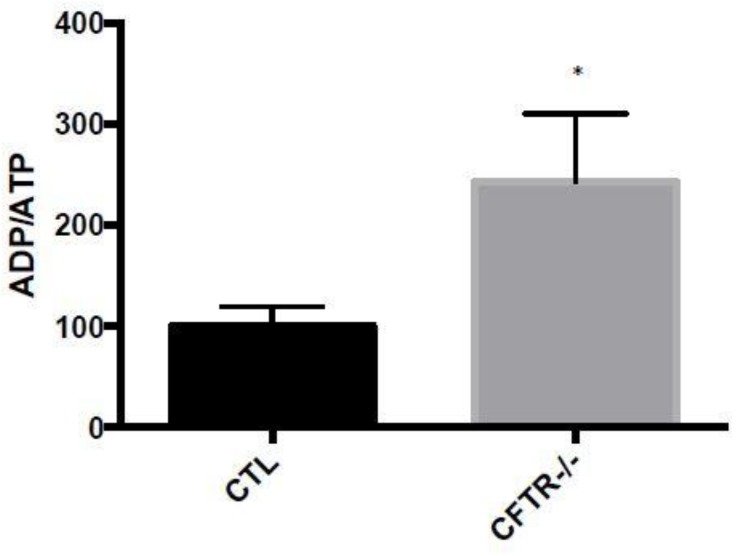
Effects of CFTR deletion on mitochondrial ADP/ATP ratio. Caco-2/15 cells were allowed to differentiate until 15 days after confluence. Then, cells were, harvested, trypsinized and homogenized. The ADP/ATP ratio was measured in isolated mitochondria by a bioluminescence assay. Values are means ± SEM of 3 independent experiments, each performed in triplicate. * *p* < 0.05 vs. CTR cells. CTR: control cells; FA: iron/ascorbate (200 μM/0.5 mM); BHT: Butylated hydroxy toluene (0.5 mM).

As the major biological function of mitochondria consists in generating cellular energy through FA β-oxidation and oxidative phosphorylation, we measured mitochondrial ADP/ATP ratio. The results showed that CFTR^-/-^ cells spontaneously exhibited a significant elevation (185%, *p* < 0.05) in ADP/ATP ratio compared to CTL cells (Figure 5).

### 3.5. Apoptosis-Related Proteins

**Figure 6:**
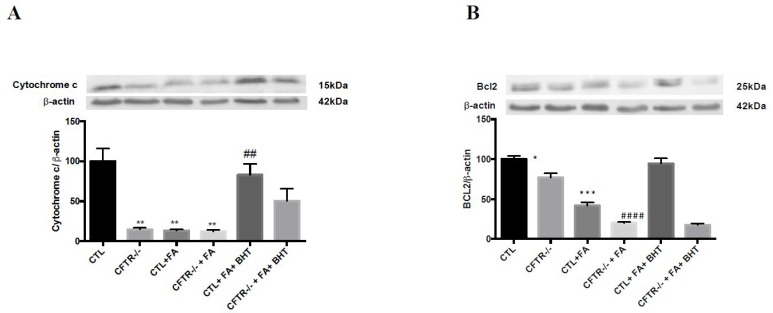
Impact of CFTR depletion on mitochondrial apoptosis-related proteins. Mitochondria were isolated from differentiated Caco-2/15 cells by ultracentrifugation. Then, protein mass of (**A**) cytochrome c and (**B**) B-cell lymphoma 2 (Bcl2) was quantified by western blotting. Values are means ± SEM of 3 independent experiments, each performed in triplicate. * *p* < 0.05, ** *p* < 0.01, *** *p* < 0.001 vs. CTL cells; *##* *p* < 0.01, #### *p* < 0.0001 vs. CFTR^-/-^ cells. CTR: control cells; FA: iron/ascorbate (200 μM/0.5 mM); BHT: Butylated hydroxy toluene (0.5 mM).

Mitochondrial cytochrome c and Bcl-2 proteins usually serve to explore apoptosis. Our experiments demonstrated that, under basal conditions, the protein expression of cytochrome c (14%, *p* < 0.01) (Figure 6A) and Bcl2 (77%, *p* < 0.01) (Figure 6B) in the mitochondria of CFTR^-/-^ cells was significantly lower than that in CTL cells. Fe/Asc administration led to deep decreases in Bcl2 protein expression particularly in CFTR^-/-^ cells (19.9%, *p* < 0.0001) (Figure 6B) and cytochrome c protein expression remained low (12%, *p* < 0.01) (Figure 6A). Pre-incubation of cells with BHT significantly restored mitochondrial expression of especially cytochrome c in CTL cells (83%). (Figure 6).

### 3.6. Lipogenesis

**Figure 7:**
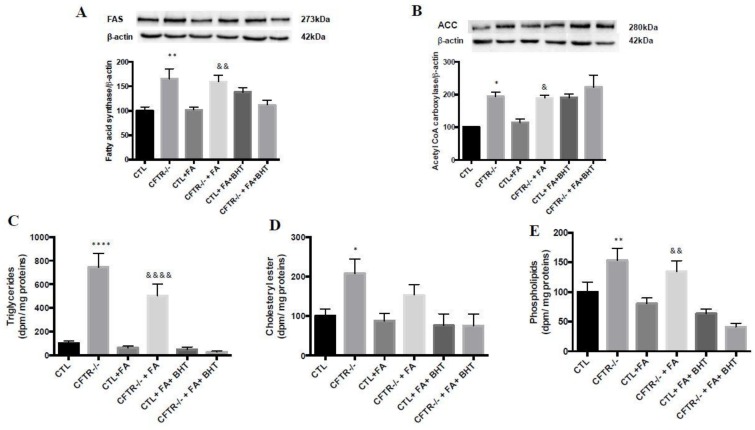
Influence of CFTR deletion on lipogenic enzymes and lipid synthesis. At the end of 15 days of differentiation, Caco-2/15 cells were harvested, trypsinized, and homogenized. Protein mass of (**A**) fatty acid synthase (FAS) and (**B**) acetyl CoA carboxylase (ACC) was analyzed by western blotting. Following a challenge cells with (^14^C)-oleic acid, lipid synthesis was assessed by quantification of (**C**) triglycerides, (**D**) cholesteryl ester and (**E**) phospholipids in basolateral medium. Values are means ± SEM of 3 independent experiments, each performed in triplicate. * *p* < 0.05, ** *p* < 0.01, **** *p* < 0.0001 vs. CTL cells; & *p* < 0.05, && *p* < 0.01, &&&& *p* < 0.0001 vs. CTL + FA cells. CTR: control cells; FA: iron/ascorbate (200 μM/0.5 mM); BHT: Butylated hydroxy toluene (0.5 mM).

As opposite regulation characterizes FA β-oxidation and the lipogenesis pathway, we examined the protein expression of lipogenic enzymes in total homogenates to test whether they were up-regulated in contrast with the down-regulated enzymes reported above in the FA catabolic process. Indeed, the results revealed that, under basal conditions, FAS (165%, *p* < 0.01) and ACC (194%, *p* < 0.05) protein expression was significantly increased in CFTR^-/-^ compared to CTL cells (Figure 7A,B). Comparable differences between CFTR^-/-^ and CTL cells were also noted in FAS and ACC in the presence of Fe/Asc. The addition of BHT was without marked effect on Fe/Asc-induced levels (Figure 7A,B).

### 3.7. Lipid Synthesis

In a second step, we challenged CTL and CFTR^-/-^ cells with oleic acid in order to evaluate their ability to synthesize lipids and lipoproteins in Caco-2/15 cells granting access to both sides of the bipolar intestinal epithelium. The results demonstrated that under basal conditions, the CFTR^-/-^ cells significantly raised the output of TG (644%, *p* < 0.0001) (Figure 7C), CE (108%, *p* < 0.05) (Figure 7D) and PL (53%, *p* < 0.01) (Figure 7E) in the basolateral medium. The addition of Fe/Asc led to a drop of lipid secretion in the two cell types. However, the export of lipids to the basolateral medium remained higher in CFTR^-/-^ cells (~5 fold of CTL values). On the other hand, BHT decreased lipid secretion, especially triglycerides, in CFTR^-/-^ and CTL cells (Figure 7C).

### 3.8. Lipoprotein Production

**Figure 8:**
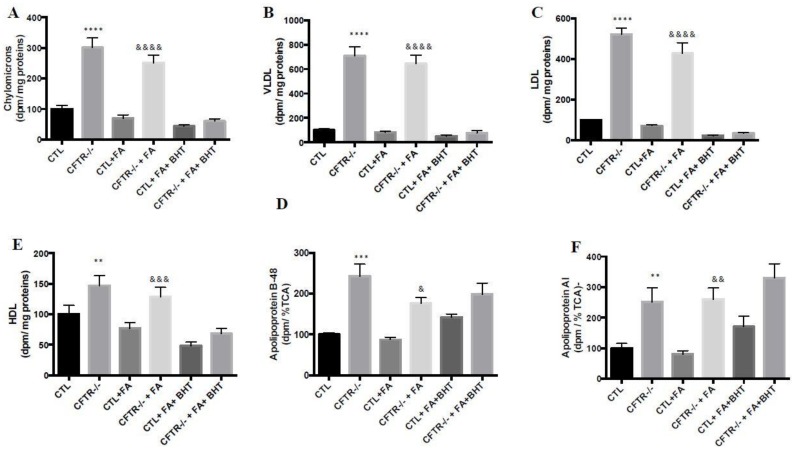
Influence of CFTR deletion on lipoprotein production and on *de novo* apolipoprotein synthesis in Caco-2/15 cells. Caco-2/15 cells were allowed to differentiate until 15 days after confluence. Then, cells were challenged with (^14^C)-oleic acid. Lipoproteins production was assessed by quantification of (**A**) chylomicrons, (**B**) very low-density lipoproteins, (**C**) low-density lipoproteins, and (**D**) high-density lipoproteins in basolateral medium. Caco-2/15 cells were challenged with (^35^S)-methionine and *de novo* apolipoproteins (apo) synthesis was assessed in cell lysate by the measurement of apos (**E**) B-48 and (**F**) A-I. Values are means ± SEM of 3 independent experiments, each performed in triplicate. ** *p* < 0.01, *** *p* < 0.001, **** *p* < 0.0001 vs. CTL cells; & *p* < 0.05, && *p* < 0.01, &&& *p* < 0.001, &&&& *p* < 0.0001 vs. CTL + FA cells. CTR. control cells; FA: iron/ascorbate (200 μM/0.5 mM); BHT: Butylated hydroxy toluene (0.5 mM).

As intestinal lipids are transported by lipoproteins, we investigated the impact of CFTR deletion on lipoprotein production. In accordance with the elevated levels, under basal conditions, CFTR^-/-^ cells were characterized by increased concentrations of chylomicrons (201%, *p* < 0.0001) (Figure 8A), VLDL (610%, *p* < 0.0001) (Figure 8B), LDL (421%, *p* < 0.0001) (Figure 8C) and HDL (47%, *p* < 0.01) (Figure 8D) compared to CTR cells. The addition of Fe/Asc resulted in a slightly reduced secretion of lipoproteins while BHT further decreased their production (Figure 8).

### 3.9. Apolipoprotein Biogenesis

Since Apos are important components of lipoproteins, their biogenesis was assessed following the incubation of CTL and CFTR^-/-^ cells with (^35^S)-methionine as a radiolabelled precursor. CFTR^-/-^ cells spontaneously synthetized Apo B-48 (143%, *p* < 0.001) (Figure 8E) and Apo A-I (151%, *p* < 0.01) (Figure 8F) in higher quantities than CTL cells. Fe/Asc reduced the *de novo* synthesis of Apo B-48 without a significant effect on Apo A-I, but BHT intensified the synthesis in both cell types.

## 4. Discussion

CFTR-mediated fluid secretion is necessary for appropriate intestinal physiology [36], but CFTR mutations lead to various intestinal manifestations [37]. The most prominent disorders in the CF gastrointestinal tract include intestinal obstruction, hyperpermeability, impaired secretion of mucus and bicarbonate, bacterial overgrowth, microflora changes, hyperacidity, dysmotility, and malignancies [38]. The current data are consistent with and support the findings of our previous study demonstrating that the simple knockout of CFTR in intestinal epithelium causes redox disequilibrium [17]. However, the present innovative work was able to highlight the mitochondrial dysfunction in response to intestinal CFTR depletion, which was underlined by the derangement of FA β-oxidation, oxidative phosphorylation, and energy production, along with apoptosis amplification. These disturbances were associated with the down-regulation of PGC-1α, a master regulator of mitochondrial biogenesis and oxidative metabolism [39]. Concomitantly, a significant stimulation was observed in lipogenesis, lipid synthesis, apo biogenesis and lipoprotein production. Overall, these original data demonstrate that CFTR deficiency influences multiple aspects of intestinal cellular and mitochondrial homeostasis.

Most cellular studies on the role of CFTR generally employed genetic manipulations that only partially reduce cellular CFTR expression. However, heterozygotes with a single CFTR mutation do not have CF, suggesting that partial loss of CFTR function could not contribute to the disease. Thus, the ZFN technique used in the present study provides a considerable advantage since it leads to the disruption of all CFTR transcripts and to the total depletion of the CFTR protein, which mimics the homozygous CF phenotype. Moreover, the genetic engineering was carried out in Caco-2/15 cells that undergo a spontaneous differentiation leading to the formation of a monolayer of cells expressing several morphological and functional characteristics of mature human enterocytes [40]. This remarkable intestinal model is not only regarded as the most convenient for the investigation of gut absorption and interactions [29], but it has been fully appropriate in our hands to explore lipid/lipoprotein homeostasis, OxS and inflammation [40]. Therefore, we are very confident that our experimental tools are fully optimal for the scrutiny of the modulatory role of CFTR in mitochondrial function.

ROS overproduction in CF is generally attributed to malabsorption of fat-soluble antioxidant vitamins [41], infection conditions [42], stimulation of NADPH oxidases through inflammation mediators [42] and a constitutive defect of GSH metabolism [43]. However, our recent studies showed that the very fact of abrogating the CFTR gene expression in intestinal cells was sufficient to induce OxS and to increase susceptibility to pro-oxidants [17]. The results of the present work go beyond this knowledge since they underscore the impact of CFTR depletion on mitochondrial redox homeostasis [44]. A potential mechanism for the pro-oxidant action of CFTR^-/-^ in mitochondria could be through the noted down-regulation of PGC-1α, a key regulator of the gene expression of antioxidant enzymes such as superoxide dismutase, glutathione peroxidase and catalase [45]. Furthermore, as PGC-1α modulates the antioxidant Keap1-Nrf2-ARE pathway [8], its decreased expression noted in our experiments could have been behind the down-regulation of Nrf2, a potent stimulator of oxidant scavenging systems. Although the fall of these powerful transcription factors may explain the mitochondrial imbalance of oxidants and anti-oxidants, additional work is needed to elucidate the underlying mechanisms for the interactions between CFTR and PGC-1α. But at the moment, all we know is that the effects of PGC-1α are likely to involve interactions with several DNA-binding transcription factors, such as CBP, the binding protein of CREB (cAMP response element binding protein), which brings PGC-1α to the regulatory regions of target genes [46]. Given the alterations of CBP expression caused by CFTR deficiency [46,47], it is possible to suggest that PGC-1α may be unable to coordinate regulation of transcription and splicing in response to signals relaying metabolic needs, thereby resulting in the dysregulation of mitochondrial biogenesis and function, as well as abnormal energy metabolism.

Nrf2 protein is considered as one of the main cellular defense mechanisms against OxS. It is central in the upregulation of endogenous antioxidant response [48]. In fact, Nrf2 activates the transcription of antioxidant enzymes by binding to the antioxidant response element (ARE) in the promoter regions of its target genes [49,50]. In our previous study, we demonstrated that CFTR KO resulted in significantly increased cellular malondialdehyde, a biomarker of OxS, and decreased glutathione peroxidase and catalase activities [17]. The present work evidenced an amplified OxS in mitochondria combined with reduced Nrf2 expression. A connection has been reported between CFTR and Nrf2. First, there is an antioxidant response element in the CFTR promoter, which was reported to recruit Nrf2 and various transcription factors. But, most importantly, the CF condition alters cAMP-mediated signaling and phosphorylation of CBP in response to CFTR loss [51], thereby reducing Nrf2 association, activity and expression [52,53].

As evidenced by our observations, mitochondrial DNA integrity seemed to be affected given the drop of OGG1 protein expression in CFTR^-/-^ cells. OGG1 is the initiating enzyme of DNA base repairs. It catalyses the excision of 8-OH-deoguanosine, the common mutagenic form of oxidized guanine, from DNA. It has been demonstrated that the alteration of cellular redox leads to the inhibition of OGG1 activity [54]. Similarly, previous studies in our laboratory demonstrated the harmful effect of OxS on mitochondria DNA integrity, essentially caused by reduced OGG1 expression [7]. Therefore, the imbalance of oxidants and anti-oxidants in CFTR^-/-^ cells could be incriminated in the abnormally low OGG1 protein expression.

We provided evidence that CFTR deletion in intestinal epithelial cells provoked mitochondrial dysfunction as highlighted by the decline of FA β-oxidation, oxidative phosphorylation and ATP-linked respiration. The reduction in these major metabolic pathways may be explained by the down-regulation of PGC-1α, which represents a powerful activator of mitochondrial biogenesis, oxidative metabolism and FA β-oxidation in many tissues [55]. Through its ability to coactivate numerous DNA-binding transcription factors, including nuclear respiratory factors peroxisome proliferator-activated receptor-α, and estrogen-related receptor-α, it induces the expression of a core genetic program needed for mitochondrial function, DNA replication, and transcription [39]. An example of the link for the regulation of the nuclear and mitochondrial genomes by PGC-1α is represented by the forced expression of PGC-1α that dramatically increases mitochondrial biogenesis and oxidative metabolism in certain tissues in vivo [39], whereas deletion of both PGC-1s has the opposite effect, severely limiting respiration in skeletal muscle or heart [56]. For its part, Nrf2 plays an important role in mitochondrial homeostasis [57,58]. Together, our data demonstrate that CFTR deletion leads to a drop of mitochondrial respiration, FA β-oxidation and energy production by mechanisms implicating PGC-1α and Nrf2.

Our experiments revealed the stimulation of lipogenic enzymes, lipid synthesis and lipoprotein production in Caco-2/15 cells with CFTR deletion. Although these data were intuitively expected given the depressed mitochondrial FA β-oxidation, the mechanisms remain unclear. Nevertheless, it should be noted that, in line with the present findings, a similar stimulatory trend was reported in previous studies using infection with short hairpin RNA interference-expressing lentiviruses, which suppressed the CFTR protein expression by 50% [29]. Importantly, phosphorylation of AMP-activated protein kinase (AMPK) may be reduced in cells with CFTR deficiency, which will lead to a decline in ATP synthesis and low ADP/ATP [59]. Such a situation would reduce phosphorylation of downstream targets of AMPK, including ACC, which would lead to increased FA synthesis. Consistent with our findings, it is reasonable to propose that intestinal malabsorption in CF is not due to intracellular processes governing lipid synthesis and CM production, but rather to luminal factors, including exocrine pancreatic insufficiency, accumulation of viscous and sticky mucus, bacterial overgrowth, ileal hypertrophy and villous atrophy, augmented intestinal permeability, reduced secretion of sodium bicarbonate, low duodenal pH, and bile acid precipitation [16].

Our work is original because precisely very few studies have focused on inflammation, OxS, lipids/lipoprotein formation, mitochondrial function and the expression of powerful transcriptional factors regulating intracellular events in the intestine of CF patients and animal models. In line with the present study, it has recently been reported that ΔF508 mice developed intestinal inflammation through increased neutrophil and macrophage infiltration, which induced aberrant activation of NF-κB in the mucosal layer of the small intestine [60]. Similarly, intestinal epithelial cells from CF patients bearing the F508del-CFTR mutation exhibited an impressive derangement of cellular proteostasis, with OxS [61]. On the other hand, regarding the formation of intestinal lipoproteins, the literature is very poor and is limited to describing the malabsorption of fats, essentially caused by digestive defects related to pancreatic lipase, bile acids and bicarbonate [62]. The impact of CFTR deficiency on intra-enterocyte processes has been very little investigated. Yet our results are in agreement with the TG concentrations and TG/HDL-C ratios above population means [63,64,65], a dyslipidemic phenotype representing a sign of premature atherosclerosis that was already reported in CF patients [66]. As to mitochondrial dysfunction in CF, several hypotheses were put forward before CFTR gene discovery to incriminate this organelle as an etiological factor for the CF pathology [6,67]. The limited studies available in the scientific literature on the role of mitochondria in CF are cited in these two reviews, which also documented a reduction in mitochondrial Complex I electron transport chain function. Moreover, characterization of mitochondrial function in airway cells homozygous for the F508del-CFTR allele revealed impairment of oxygen consumption, ΔΨ generation, adenine nucleotide translocator-dependent ADP/ATP exchange and both mitochondrial Complex I and IV activities [68]. However, there have been no additional studies to determine the full set of mitochondrial alterations in the gut of CF patients and animal models.

While little information is available for PGC-1α, further observations are provided in the literature on the role of Nrf2 and lipoprotein metabolism. Mice with Nrf2 knockout exhibit high serum TG/lipids and enhanced VLDL secretion in association with induced hepatic apo B and microsomal triacylglycerol transfer lipoprotein [69]. Accordingly, Nrf2 deficiency retards atherosclerotic lesion development [70] and aggravates atherosclerosis in LDLR^-/-^ mice [71,72]. However, controversial findings were also reported since Nrf2 deficiency alleviates atherosclerosis in hypercholesterolemic ApoE^-/-^ mice [73,74]. Therefore, additional studies are warranted in order to define the contribution of Nrf2 to intestinal lipoprotein metabolism and cardiovascular diseases, especially in response to CFTR deficiency that affects the expression of Nrf2.

## 5. Conclusions

The current work provides a gathering body of evidence related to the role of CFTR in the preservation of mitochondrial dysfunction in enterocytes. Clearly, CFTR deficiency leads to dysfunction in mitochondrial energy metabolism as exemplified by the deterioration of FA β-oxidation, oxidative phosphorylation and ATP production in intestinal epithelial cells. The data also highlight the adverse effects of CFTR deletion on the expansion of cellular lipogenesis and lipoprotein output. Although the findings pointed out the implication of OxS and robust transcription factors in the disruption of cellular and mitochondrial homeostasis, further studies are needed to delineate the specific mechanisms.

## Figures and Tables

**Table 1:** CFTR knockout and Caco-2/15 cell integrity.

VIABILITY CRITERIA	Control Cells	CFTR^-/-^ Cells
Trypan blue exclusion	>96%	>96%
Transepithelial resistance (Ω × cm^2^)	100 ± 20	96 ± 14
Sucrase activity (UI/g proteins)	100 ± 21	94 ± 15
Villin protein expression (% of controls)	100 ± 11	102 ± 11
Occludin protein expression (% of controls)	100 ± 6	105 ± 5

Following CFTR silencing by the ZFN technique, Caco-2/15 cells were allowed to differentiate for 15 days. The cellular integrity was assessed using trypan blue exclusion (as a viability marker) and also by the determination of transepithelial resistance and occludin (as indicators of cell permeability and epithelial barrier function, respectively), as well as the expression of sucrase activity and villin protein mass (as markers of cell differentiation). Values are means ± standard error of the mean (SEM) of 3 independent experiments, each performed in triplicate.

## Abbreviation

ACADL	acyl-CoA dehydrogenase long-chain
ACC	acetyl CoA carboxylase
AMPK	AMP-activated protein kinase
ARE	antioxidant response element
BHT	butylated hydroxy toluene
CF	cystic fibrosis
CPT1	carnitine palmitoyltransferase
CTL	controls
FAS	fatty acid synthase.
Fe/Asc	iron-ascorbate
GSH	glutathione
Nrf2	nuclear factor like 2
OGG1	8-oxoguanine-DNA glycosylase
OxS	oxidative stress
PGC-1α	peroxisome proliferator activated receptor gamma coactivator-1-alpha
PPARα	peroxisome proliferator-activated receptor alpha
ROS	reactive oxygen species
ZFN	zinc finger nuclease
